# Using DenseFly algorithm for cell searching on massive scRNA-seq datasets

**DOI:** 10.1186/s12864-020-6651-8

**Published:** 2020-12-16

**Authors:** Yixin Chen, Sijie Chen, Xuegong Zhang

**Affiliations:** 1 Department of Automation, MOE Key Laboratory of Bioinformatics; Bioinformatics Division and Center for Synthetic & Systems Biology, BNRist, Tsinghua University, Beijing, 100084 China; 2 School of Life Sciences, Tsinghua University, Beijing, 100084 China

**Keywords:** DenseFly, Locality sensitive hashing, scRNA-seq, Cell searching

## Abstract

**Background:**

High throughput single-cell transcriptomic technology produces massive high-dimensional data, enabling high-resolution cell type definition and identification. To uncover the expressional patterns beneath the big data, a transcriptional landscape searching algorithm at a single-cell level is desirable.

**Results:**

We explored the feasibility of using DenseFly algorithm for cell searching on scRNA-seq data. DenseFly is a locality sensitive hashing algorithm inspired by the fruit fly olfactory system. The experiments indicate that DenseFly outperforms the baseline methods FlyHash and SimHash in classification tasks, and the performance is robust to dropout events and batch effects.

**Conclusion:**

We developed a method for mapping cells across scRNA-seq datasets based on the DenseFly algorithm. It can be an efficient tool for cell atlas searching.

## Background

Single-cell RNA sequencing (scRNA-seq) technologies measure transcriptional profiles of individual cells, enabling high-resolution approaches for cell-type (subtype) definition and offering in-depth insights into cell-to-cell variations [1–3]. High-throughput scRNA-seq data is accumulating at massive scales [4]. For instance, Han et al. [5] and the Tabula Muris Consortium et al. [6] have published two mouse scRNA-seq datasets, each with ~ 100,000 cells characterized by the expression of thousands of genes. The ongoing Human Cell Atlas (HCA) project is aiming to provide the profiles of all human cell types as a reference for future studies and is already producing massive single-cell omics data for many human tissues and organs [7, 8].

The accumulation of scRNA-seq data allows the comparative study of cells, which is a basic step in the utilization of cell atlas data in the future. Given a set of query cells, we need to search against the curated reference cells collected from HCA datasets or other datasets, identify the most similar cells in the reference, and infer the properties of the queries. As the query and the reference cell profiles are a vast collection of gene expression vectors of very high dimensionality (e.g., up to ~ 10,000 gene expression features for millions of reference cells), the efficiency of traditional tree-based data searching methods will be challenged in time memory consumption. There have been several researches mapping/searching cells across different datasets such as scmap [9], CellAtlasSearch [10] and comparisons [11] between methods are available.

Locality Sensitive Hashing (LSH) is a probabilistic algorithm for finding similar elements from a large database. LSH encodes a high-dimensional data point into a binary vector, and the similarity between points is obtained by comparing the common elements of the encoded vectors. CellAtlasSearch [10] is the first method using LSH for cell searching. It provides a web-interface for cell searching against single-cell or bulk RNA-seq dataset. However, its methods are not described in detail in its original paper and the source code is not freely available online. CellFishing.jl [11] is another implementation of cell searching in LSH with a systematic performance evaluation. CellFishing.jl conducted several substantial experiments including mapping cells across different batches, different species, and different protocols.

In this work, we adopted the DenseFly algorithm [12] for the cell searching problem and conducted a series experiments for different scenarios to compare it with existing methods. DenseFly algorithm is a variant of classical LSH. Its encoding scheme is inspired by the fruit fly’s odor circuit. Our experiment results indicated DenseFly outperforms benchmark methods in cell type matching accuracy (Cohen’s Kappa [13]) and is resistant to typical scRNA-seq data noises such as dropout events [14, 15] and batch effects [16].

## Results

### Cell type identification performance

**Fig. 1:**
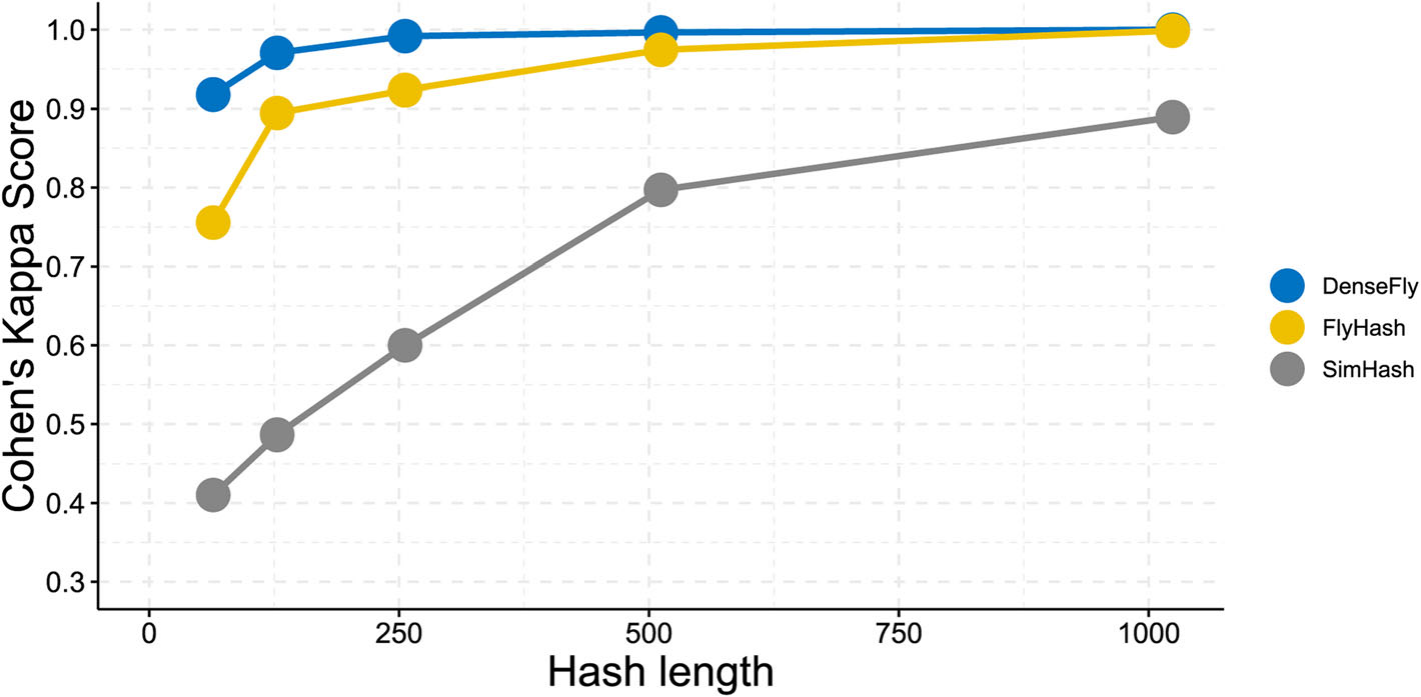
The result of the cell type identification experiment. The x-axis is the hash length while the y-axis is the average Cohen’s Kappa score given by the five-fold cross-validation. The performance of all algorithms improved as the hash length becomes larger. The figure indicates that DenseFly outperforms FlyHash and SimHash under all chosen hash length conditions, and DenseFly still reaches a high score even with a relatively shorter hash length. The experiment proves DenseFly’s feasibility on the scRNA-seq data similarity search

We compared the classification performance of DenseFly algorithm with the benchmark methods FlyHash and SimHash on SIM I dataset (Fig. 1). The results show that DenseFly achieves higher Cohen’s Kappa under all tested parameter conditions. Another fly-inspired algorithm FlyHash has weaker classification performances, while the SimHash performs significantly worse than DenseFly and FlyHash.

This experiment is a self-mapping test. 20% random samples from SIM I are given as queries and the three algorithms report the label of the most similar cell that lies in the rest as the classification result. We used several hash lengths (*k* = 64, 128, 256, 512, 1024) and left other parameters unchanged: the sampling rate *α* was 0.1, the embedding size of FlyHash and DenseFly was 20 times of hash length (m = 20).

### Resistance to batch effects

We tested whether DenseFly has the resistance to the batch effect on SIM II dataset. As shown in Table 1, SIM II dataset has two subsets: Batch 1 and Batch 2, which are simulated to be the same cell types but from different experiment batches. We tried to map cells from Batch 1 to Batch 2 and vice versa and compared the classification performance with SIM I experiments without batch effects. The same five-fold cross-validation is used to get average Cohen’s kappa scores too. The parameters used in these experiments is the same as SIM 1: k = 64, 128, 256, 512, 1024; α = 0.1; and m = 20.

**Table 1:** Time consumption per hundred queries (unit: seconds)

Reference size	SimHash	FlyHash	DenseFly
200	0.012	0.012	0.012
400	0.017	0.018	0.018
800	0.025	0.026	0.024
1000	0.026	0.029	0.028
2000	0.040	0.042	0.041
4000	0.097	0.105	0.099
8000	0.156	0.157	0.158

**Fig. 2:**
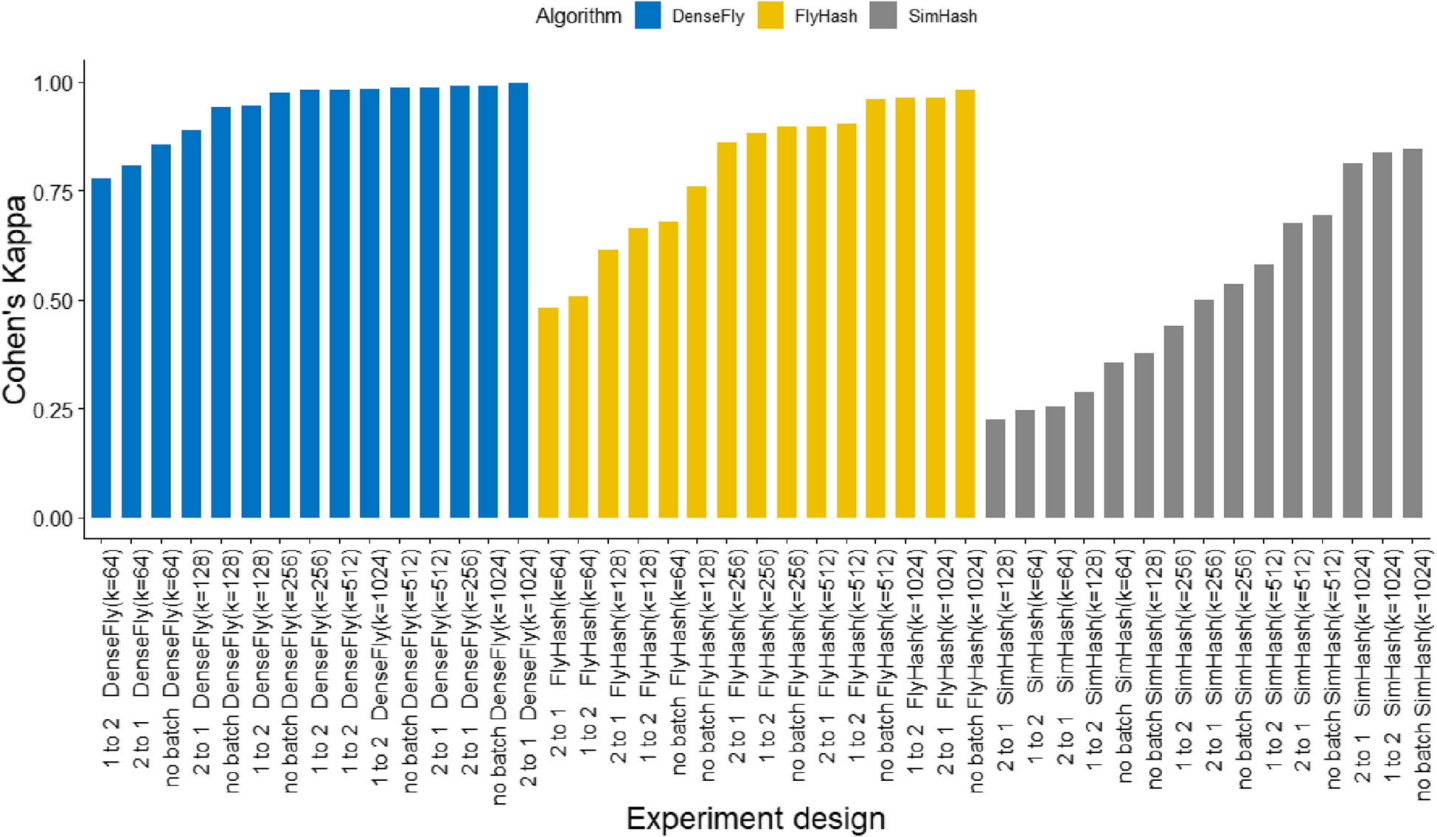
The batch effect experiment bar plot. The x-axis lies the name of experiment designs and the y-axis is the Cohen’s Kappa score. The blue bars represent experiments conducted on DenseFly, yellow bars represent experiments on FlyHash, and gray bars represent SimHash’s results. DenseFly shows the best batch-proof performance among the three methods while SimHash is the worst. FlyHash achieves similar performance to DenseFly but it relies on large hash lengths. In general, the cross-batch mappings have lower scores than no-batch mappings, except for DenseFly’s results. There is no significant difference between “1 to 2” and “2 to 1”, which accords with our simulation settings.

The mapping performance in Fig. 2 shows that batch effect does not affect DenseFly significantly while the other two methods are less robust when batch effects exist. Compared with FlyHash, DenseFly achieves higher scores with lower hash lengths. It is also noteworthy that DenseFly achieves high performances regardless of batch effect – it achieves even higher scores than the no-batch group. These comparisons on simulation datasets indicate that DenseFly has high resistance to the batch effects.

### Resistance to dropout events

We also tested whether DenseFly has high resistance to dropout events on the SIM III dataset. As shown in Table 1, a series of dropout rates from 0% to ~ 50% are considered. The same five-fold cross-validation is used to get average Cohen’s kappa scores with the parameter settings: k = 32, 64, 128, 256; α = 0.1; and m = 20.

**Fig. 3:**
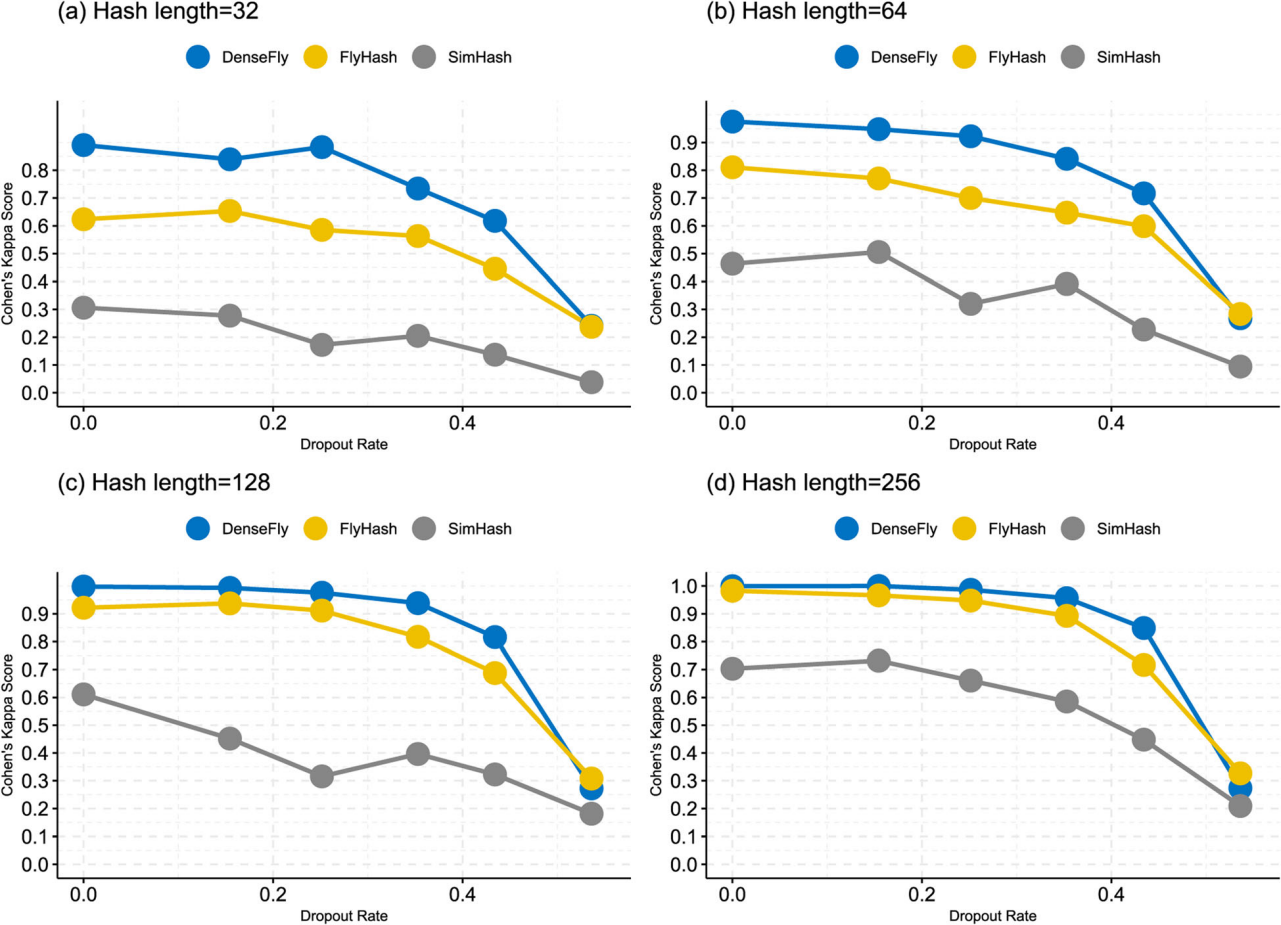
The result of dropout experiments**. a-d** show the Cohen’s Kappa score changing with dropout rate under different hash length (32, 64, 128, 256). The x-axis of each figure is the dropout rate and the y-axis of each figure is Cohen’s Kappa score. It is reasonable that the performances of three algorithms all decrease as the dropout rate increase. We can see that DenseFly always outperforms others and has a stable ‘platform’ range where Cohen’s Kappa score decreases slowly when the dropout rate is small, particularly when hash length = 256. The experiments show DenseFly is robust when the dropout event occurs. It should be explained that the original data without dropout has 45% zero elements in the expression matrix, meaning that SIM III-5 dataset (dropout rate = 53.6%) is extremely sparse (over 98% elements is zeros), so all algorithms perform poorly because little information remained in the dataset

The experiment results in Fig. 3 show that DenseFly has the highest dropout-proof ability, and all methods’ performances decrease significantly as the dropout rate increases. As hash length increases, FlyHash achieves similar performances to DenseFly but still, SimHash lags significantly.

### Time consumption and scalability

The growing scale of single-cell RNA-seq datasets sets a strict requirement on time complexity. We used python to implement the algorithms and the queries are computed in serial with CPU and recorded the time consumption per 100 queries when reference cell number varies (Table 1). The results show that our implementations (k = 128; α = 0.1; m = 20) of SimHash, FlyHash and DenseFly take equal time to finish to queries and time consumption grows linearly as the reference size grows. Since all queries run independently, a parallel optimization can be achieved easily where a query results will be returned from an atlas-level database in seconds.

## Discussion

The extraordinary performances shown in this work give strong evidence that DenseFly is a better alternative to SimHash, the method already used in cell searching. Compared with traditional LSH implementations, DenseFly ensembles multiple random samples from the original feature space to build up intermediate activations. This procedure helps to avoid gene dropout events in scRNA-seq data because the dropped genes may not lie in the sampled fraction. Note that scRNA-seq data is centralized first in DenseFly and FlyHash, the encoding schemes binarize the activations and partially eliminates the batch effects. This explains why DenseFly and FlyHash are so resistant to batch effects.

Although DenseFly and FlyHash perform well on simulated batch effects and dropout events, it doesn’t mean that the missing value imputation methods and normalization methods for scRNA-seq are not necessary. We expect further studies to add these preprocessing steps and infer that DenseFly and FlyHash would have even better performance.

It is Drosophila’s olfactory system that inspires the DenseFly and FlyHash algorithms. The two algorithm’s data structure can be a good analog to the olfactory circuits. The structure in fly’s olfactory is not unique, and similar neural cell compositions and structures can also be found in other vertebrate brain regions [11]. It is estimated that a trained human nose can recognize up to a trillion smells [13]. Therefore, it is interesting to know whether DenseFly or its future variants could have a very huge model capacity. It is known that human beings have approximately 200 main cell types and new sub-types are constantly revealed by projects like Human Cell Atlas. It remains to be seen whether these bionic algorithms could play more important roles in recognizing new cells (sub) types and yielding new biological knowledge.

## Conclusions

Cell searching is playing building block roles in identifying similar cells, defining cell types, and revealing cellular relationships from atlas-scale datasets. The simulation experiments have shown that DenseFly-based cell type identification out-performs FlyHash and SimHash in Cohen’s kappa score, and the performance is robust when dropout or batch effect noises exist. The different hashing schemes of the LSH implementations indicate suitable structures and feature processing steps should be chosen for specific tasks. It is probably the random sampling and shuffling that assist DenseFly to adapt to the sparse and noisy scRNA-seq data. This study provides a new solution to LSH-based cell atlas searching. Though only simulated scRNA-seq data is used in the experiments, the simulations are representative and capture the main characteristics of the data. More experiments on real data should be done in the future to further endorse the new solution’s application. There is also a need for more advanced implementations, such as parallel computing, or GPU support, to further speed up DenseFly algorithm. We hope researchers and developers in the cell search field could pay more attention to DenseFly and build up powerful searching tools with it.

## Methods

### The cell searching problem of scRNA-seq data

Single-cell RNA-seq data are the measurement of the expression of thousands or tens of thousands of genes in each single cell. A reference dataset can contain data of millions of cells or more. Each cell is a gene expression vector or a column in a data matrix. The task of cell searching is to find the cell in the reference dataset that is the most similar to a query cell in the gene expression. It is also called cell mapping in some context, which usually concentrates on the mapping of the query cell to a certain cell type or subtype instead of finding the most similar cell.

We centralize the gene expression of each cell by subtracting the mean expression of all genes in the cell from the expression of each gene. The same processing is also applied to the query data. For convenience, the centralized values are still referred to as “gene expression” when there is no confusion.

There are different ways to calculate gene expression values from the original sequencing data, such as read counts, UMI-counts, RPKM and TPM. The methods we studied can be applied to any of these types as long as both the query samples and reference samples use the same way of calculation.

### LSH-based similarity searching

Locality sensitive hashing (LSH) is a similarity searching algorithm for high dimensional data where classical tree-based searching methods for lower dimensional data fail due to their prohibitive time and memory consumption. LSH encodes input vectors to a bit array (a binary hash vector of lower dimension usually), in which two similar input vectors have a higher probability of sharing more common bits. As a general high-dimensional data searching method, LSH has been applied in many fields like image similarity identification [17], duplicated document detection [18], etc.

LSH have different implementations, some of which have been employed for cell searching. In this article, we studied the cell search performance of three representative implementations: SimHash [19], FlyHash [20], and DenseFly [12]. SimHash has been used by CellFishing.jl for cell searching tasks. FlyHash and DenseFly are new methods inspired by Drosophila’s olfactory neural system and have not been adopted for the task before. All three different implementations map a vector of *d* dimensions to a binary *mk*-dimensional vector (i.e. the hash length equals to *mk*), but the hashing function designs are different. Here we use the product of two parameters *m* and *k* instead of one parameter to denote the number of projections to make the description compatible for the 3 methods.

After converting all high-dimensional vectors into the hash vector of dimension *mk*, the reference database to be searched against is converted to a highly compressed hash table ordered using the Hamming distance. Searching for nearest neighbors of a query vector can be efficiently implemented by finding the hash value with the minimal Hamming distance through the table.

#### Hash function of SimHash

SimHash [19] is a classical implementation of LSH for nearest neighbor searching proposed in 2002 by Moses S. Charikar, which is used by CellFishing.jl for cell searching. We employed SimHash as a baseline method in this study. Its basic idea is: Given an input vector of length *d* (i.e. the vector contains *d* elements), generate *mk* random projection vectors of length *d* (We call *mk* the as the **embedding size**)*.* Each element of the random projection vector is drawn i.i.d. from a distribution Normal(0, 1). For each random projection vector, calculate the dot product of the input vector and random projection vector, and take the sign indicator (positive or negative) of the dot product as one bit of hash value. The final hash value (hash vector) is obtained by concatenating all bits produced by the random projection vectors.

1 $$ {Hash}_p\left(\ \overset{\rightharpoonup }{x}\ \right)=\left\{\begin{array}{c}1\kern0.5em \left(\  if\ \overset{\rightharpoonup }{x}\cdotp \overset{\rightharpoonup }{r_p}\ge 0\ \right)\\ {}0\kern0.5em \left(\  if\ \overset{\rightharpoonup }{x}\cdotp \overset{\rightharpoonup }{r_p}<0\ \right)\end{array}\right.,p=1,\cdots, mk $$

2 $$ Hash\left(\overset{\rightharpoonup }{x}\right)=\left[{Hash}_1\left(\overset{\rightharpoonup }{x}\right),{Hash}_2\left(\overset{\rightharpoonup }{x}\right),\cdots, {Hash}_{mk-1}\left(\overset{\rightharpoonup }{x}\right),{Hash}_{mk}\left(\overset{\rightharpoonup }{x}\right)\right] $$

#### FlyHash and DenseFly

FlyHash was proposed by Dasgupta et al. in 2017 [20] and the improved version DenseFly was proposed in 2018 [12]. DenseFly is reported to outperform both FlyHash and SimHash in metrics including mean average precision, the area under the precision-recall curve.

Given an input vector of length d, a random sample of elements with a **sampling rate** *α* is taken from all *d* elements of the input vector. Then both FlyHash and DenseFly sum the chosen elements as one activation. The algorithms repeat these steps *mk* times to get *mk* activation values, which are intermediate results to get final hashing values.

3 $$ {a}_i\left(\overset{\rightharpoonup }{x}\right):= Sum\  of\ \left\lfloor \alpha d\right\rfloor\ chosen\ components,i=1.. mk $$

Unlike in SimHash and other traditional LSH implementations, the embedding size *mk* here is usually set high (e.g., *mk > d*) so that the information is well captured. FlyHash and DenseFly differ in their ways of treating these activations. FlyHash uses a winner-take-all (WTA) scheme to generate a hash value from *mk* activations. It first shuffles the input vector’s elements *m* times and takes the first *k* elements (*k* is also known as the **hash size** of the WTA factor) in each shuffling group.

4 $$ {\displaystyle \begin{array}{ccc}{\mathrm{Shuffle}}_{\mathrm{j}}& =& \mathrm{a}\ \mathrm{subset}\ \mathrm{from}\ \left\{{a}_1\left(\overset{\rightharpoonup }{x}\right),{a}_2\left(\overset{\rightharpoonup }{x}\right),\dots, {a}_{mk}\left(\overset{\rightharpoonup }{x}\right)\ \right\}\\ {}& & \mathrm{where}\ \left|{\mathrm{Shuffle}}_{\mathrm{j}}\right|=k\  and\ j=1..m\end{array}} $$

Then it applies a maximal value indicator for each k-element shuffling group. The maximal value indicator encodes one shuffling group into a one-hot vector of length *k* with a single 1 at the index with maximal value. For instance, if k = 5 and the shuffling group is [− 3, 1, 2, 4, − 1], the group is encoded as [0, 0, 0, 1, 0]. The *mk*-bit hash value is obtained by concatenating all *m* one-hot vectors. If, for example, we have 3 shuffling groups [− 3, − 2, 1, 0, 5], [− 1, 2, 1, 0, 7] and [0, − 2, 0, 0, 1], FlyHash obtains the *mk*-dimensional hash vector as [0, 0, 0, 0, 1, 0, 0, 0, 0, 1, 0, 0, 0, 0, 1].

DenseFly uses a different way to get the hash vectors. In the k-element shuffling groups, positive elements are encoded as 1 and other values are encoded as 0. For instance, the shuffling group [− 3, 1, 2, 4, − 1] is encoded as [0, 1, 1, 1, 0]. The returned vector is not one-hot and is denser. Similarly, a *mk*-bit hash value is also obtained by concatenating all *m* binary vectors. For the above example of 3 shuffling groups [− 3, − 2, 1, 0, 5], [− 1, 2, 1, 0, 7] and [0, − 2, 0, 0, 1], the *mk-*dimensional hash vector will be [0, 0, 1, 0, 1, 0, 1, 1, 0, 1, 0, 0, 0, 0, 1]. For convenience, we call the hash vector of *mk* dimension as the “long hash”.

#### Multi-probing

A “pseudo-hash” procedure is adopted to obtain a “short hash” of only *m* dimension. From the *m* shuffle groups obtained with (4), we sum up all the activation values of the *k* elements in a group as the activation of the group. If the summed activation is greater than zero, we encode the group as 1, and otherwise 0. In this way, we obtain the *m*-dimensional short binary hash vector. In the above example of the 3 shuffling groups, the short hash vector we obtain is [1, 1, 0]. The set of long hash vectors can be taken as a high-resolution representation of the original data, and the set of short hash vectors can be taken as a highly-abstractive low-resolution representation of the original data.

Using the *m* dimensional short hash table to represent a reference database ensures high efficiency in the searching procedure, but there are two situations we need to consider. One is that in the original vector space is of very high dimension, such as the situation of scRNA-seq data, samples in some areas of the original space may be very sparse even when there are millions of samples. This can result in a situation that the most similar cells may have different hash vectors. In that case, we may miss the true target if we only search for the vector with the same hash values. On the other hand, for some dense regions in the sample space, the same short hash vector may represent many samples in the original space. In that case, finding only the matched hash vector for a query doesn’t identify the real nearest target. Both these two situations are typical in scRNA-seq data as some cell types or subtypes are very sparse but some can be very dense.

We use the multi-probing strategy using the long and short hashes to deal with these difficulties. For a query sample, instead of trying to find the identical or closest short hash vector in the reference hash table, we first find all reference cells that are within a given radius from the query cell in the short hash space (if empty, the radius gets double). These cells are taken as candidate matches in the searching. These candidate matches can contain multiple reference cells, but they are of a much smaller set of samples comparing to the whole reference set. We then do the searching of the nearest cell in the long hash table of the candidate targets. This gives high-resolution and ensures that the best target can be found. This two-step strategy guarantees both computational efficiency and searching precision.

#### Parameter selection

The embedding size m·k, and the sampling rate α, are associated with the model performance. The experiments in Figs. 1, 2 and 3 indicate a wide range of embedding sizes and α around 0.1 work well. We suggest fine-tuning parameters according to the tolerance to inaccuracy and the demand of efficiency. if ground-truth cell type is supplied.

### Simulated single-cell RNA-seq datasets

The gene expression levels in single-cell RNA sequencing data contain high technical noises. Typically, there can be thousands of genes being detected as expression in each cell, with all remaining genes detected as zero-expression. These seemingly zero-expression genes include not only unexpressed genes but also genes that are expressed but not captured. The latter situation is called “dropout event”. It is a major cause of noise in scRNA-seq data and the proportion of dropout genes can be higher ~ 70% in some data. This phenomenon makes scRNA-seq data highly sparse.

Another major problem in scRNA-seq data is batch-effects. Due to many technical and biological restrictions, it is not possible to obtain a large-scale reference atlas in a single experiment batch. The query data are of course not from the same experiment with the reference data. The major reason for using scRNA-seq technology to study single cells is because of the pervasive existence of cell heterogeneity even the cells are of the same tissue. Normalizing batches to remove batch effect is difficult as it is hard to distinguish biological variation from technical noise. Therefore, it is highly desirable to perform cell searching between different batches.

These two issues are major challenges to the cell searching task. As the LSH algorithms especially the DenseFly algorithm can effectively and efficiently preserve the similarity relation of high-dimensional data in the hash space, we adopt the algorithm on this task. We designed a series of simulation data to mimic different situations of scRNA-seq data and used them to evaluate the suitability and performance of the three types of LSH algorithms. Simulation data allowed us to experiment on well-controlled different degrees of noise and batch effects.

We designed three artificial scRNA-seq datasets using the R packages splatter [21] for three simulation experiments. Splatter generates artificial RNA-sequencing read count matrix by sampling from Gamma-Poisson distributions, whose location and scaling parameters are adjustable to mimic real data. We can model the dropout event and the batch effect well with this tool.

In the first simulation (SIM I), a dataset is created for a basic test of the cell type identification ability of the different methods. We generated 2000 cells of 5 types. Each cell has 10,000 gene expression values measured. We randomly choose a fraction from the cells and use the remaining cells as the reference. We map the chosen cells back to the reference and measure how the mapping result agrees with the truth.

A more difficult task is designed in the second simulation (SIM II). Data points come from two batches of measurements. We also generated 2000 simulated cells, each with 10,000 genes, and also set the cells to be of 5 cell types. The first 1000 cells and the second 1000 cells are of two simulated batches, using the feature provided by splatter.

The third simulation (SIM III) contains 6 datasets with dropout rate ranging from 0 to 53.60%. Each dataset contains 2000 simulated cells, each with 10,000 gene expressional features. Five cell types are simulated. The 6 datasets cover a range of dropout rates from 0% (no dropout events) to 53.6% (heavily dropped out).

The simulation details of the three datasets are listed below in Table 2.

**Table 2:** Summary of simulation parameters

Dataset	Note	# cells	# genes	# cell types	Dropout rate
SIM I	–	2000	10,000	5	0%
SIM II	Batch 1	1000	10,000	5	0%
SIM II	Batch 2	1000	10,000	5	0%
SIM III	SIM III-0	2000	10,000	5	0%
SIM III	SIM III-1	2000	10,000	5	15.44%
SIM III	SIM III-2	2000	10,000	5	25.15%
SIM III	SIM III-3	2000	10,000	5	35.28%
SIM III	SIM III-4	2000	10,000	5	43.41%
SIM III	SIM III-5	2000	10,000	5	53.60%

Simulation parameters of SIM I, SIM II, and SIM III. Cell numbers, gene numbers, cell type numbers, and dropout rates are taken into considerations. More detail about the datasets can be found in Additional file 1

### Evaluation of performance

We evaluated the performance of the similarity search in a supervised way. The simulated single-cell expression datasets have a cell type label for each cell. Hence, we view the task as a performance evaluation of multicategory classification.

Given a query cell, though the algorithm returns multiple near neighbors, we used the nearest neighboring cell type as the mapped cell type, i.e. the classification result.

#### Cohen’s kappa

Cohen’s Kappa measures the agreement between 2 raters that classify N items into C categories [13]. When one rater is the ground truth, Cohen’s Kappa is a metric evaluating binary or multicategory classification algorithms. In this work, we adopted this metric in the cell type identification task from CellFishing.jl [11].

The scores of Cohen’s Kappa range from − 1 to 1, where 1 indicates the classification results and the ground truth are in complete agreement, 0 indicates no agreement, and negative values mean worse than random assignment. Compared with the classification accuracy, Cohen’s Kappa is more useful when samples of different classes are imbalanced because it removes the chance agreements.

Given a n-by-n confusion matrix M (classification results of n samples), the Cohen’s Kappa score is calculated by the following formula:

5 $$ Cohe{n}^{\prime }s\  Kappa=\frac{P_0-{P}_{\mathrm{e}}}{1-{P}_{\mathrm{e}}} $$

where $$ {P}_0=\frac{\sum_{i=1}^n{M}_{ii}}{\sum_{i=1}^n{\sum}_{j=1}^n{M}_{ij}} $$, and $$ {P}_e=\frac{\sum_{a=1}^C\left(\ {\sum}_{j=1}^n{M}_{aj}\cdotp {\sum}_{i=1}^n{M}_{ia}\ \right)}{{\left(\ {\sum}_{i=1}^n{\sum}_{j=1}^n{M}_{ij}\ \right)}^2} $$ .

#### Cross-validation

We used a k-fold (k = 5 in our experiments) cross-validation to evaluate Cohen’s kappa for every experiment shown in Figs. 1, 2 and 3. In each cross-validation round, we randomly divided a collection of cells into k subsets, use k-1 subsets as the training set, and calculate Cohen’s kappa with the other one test dataset.

1. Randomly choose 20% of the samples in the dataset to form the query set and leave the rest as the reference set.
2. Build models with the reference set and get the binary table of the reference set
3. Map each chosen cell to reference cells by finding the nearest neighbor in the reference sample set with the hamming distances.
4. Get the mapped cell type of each sample in the query set, compare it to the real cell type and calculate the Cohen’s Kappa
5. Repeat the steps above five times and obtain five scores for five kinds of division on the dataset
6. Return the average value of the five scores as the final CV result.

## Supplementary information

**Additional file 1.** Details_of_simulation_datasets.pptx describes the details of single-cell transcriptomic data simulation and gives the visualization of the datasets based on the dimensionality reduction algorithms.

## Data Availability

The R code scripts for single-cell transcriptomics data simulation and python code implementing the algorithms are available at https://github.com/ XuegongLab/DenseFly4scRNAseq.

## Abbreviations

scRNA-seq: single-cell RNA sequencing
LSH: locality sensitive hashing
