# Supplementary material for: Using DenseFly algorithm for cell searching on massive scRNA-seq datasets
Source: BMC Genomics. 2020 Dec 16;21(Suppl 5):222. doi: 10.1186/s12864-020-6651-8 (PMC7739457; doi:10.1186/s12864-020-6651-8)
Supplement: Supplementary file 1 — Additional file 1. Details_of_simulation_datasets.pptx describes the details of single-cell transcriptomic data simulation and gives the visualization of the datasets based on the dimensionality reduction algorithms. [file 12864_2020_6651_MOESM1_ESM.pptx]

## Slide 1
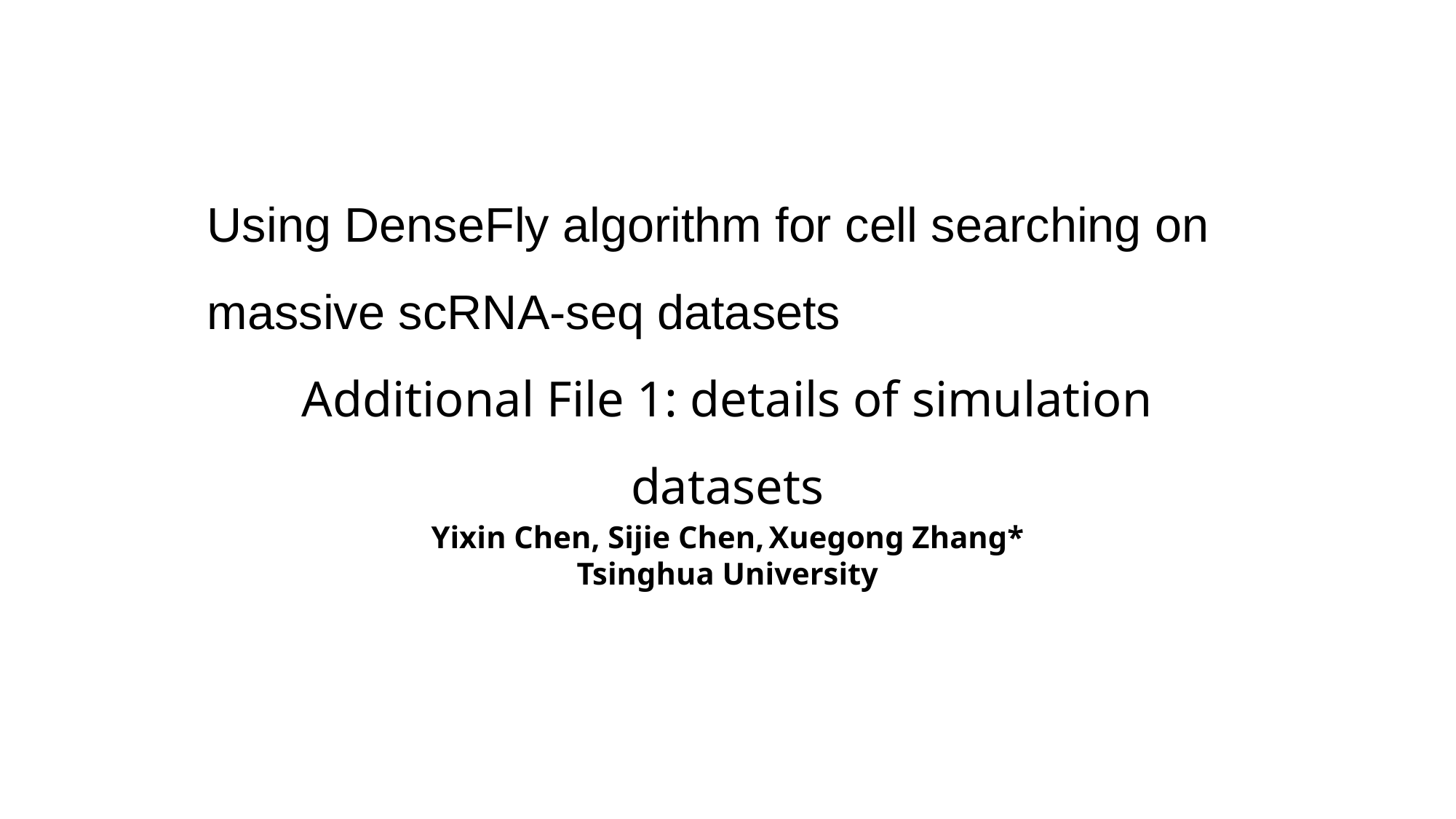

Using DenseFly algorithm for cell searching on massive scRNA-seq datasets
Additional File 1: details of simulation datasets
Yixin Chen, Sijie Chen, Xuegong Zhang*
Tsinghua University

## Slide 2
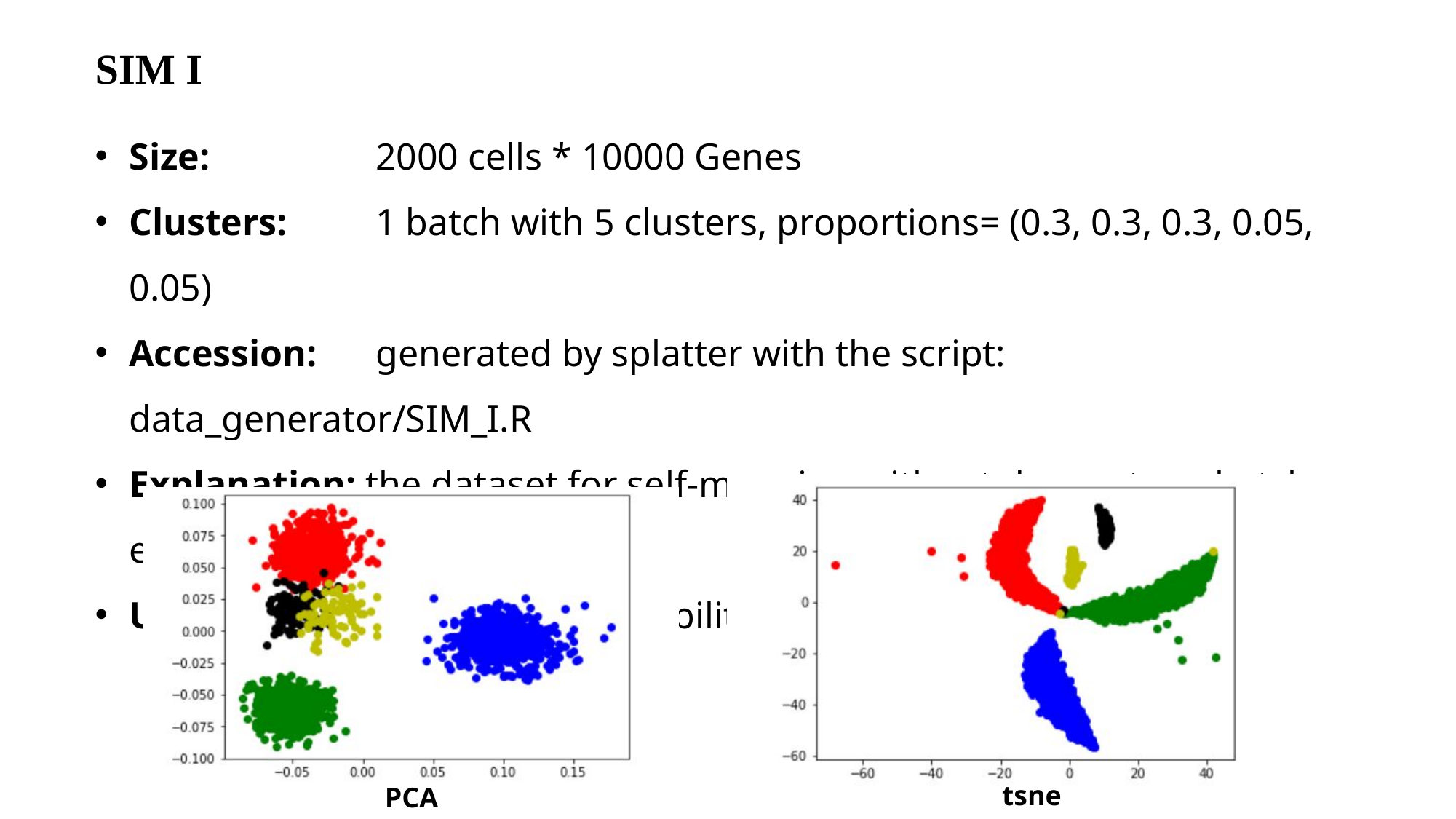

SIM I
Size:		 2000 cells * 10000 Genes
Clusters:	 1 batch with 5 clusters, proportions= (0.3, 0.3, 0.3, 0.05, 0.05)
Accession:	 generated by splatter with the script: data_generator/SIM_I.R
Explanation: the dataset for self-mapping without dropouts or batch effects
Usage:	 to prove the feasibility of a method
tsne
PCA

## Slide 3
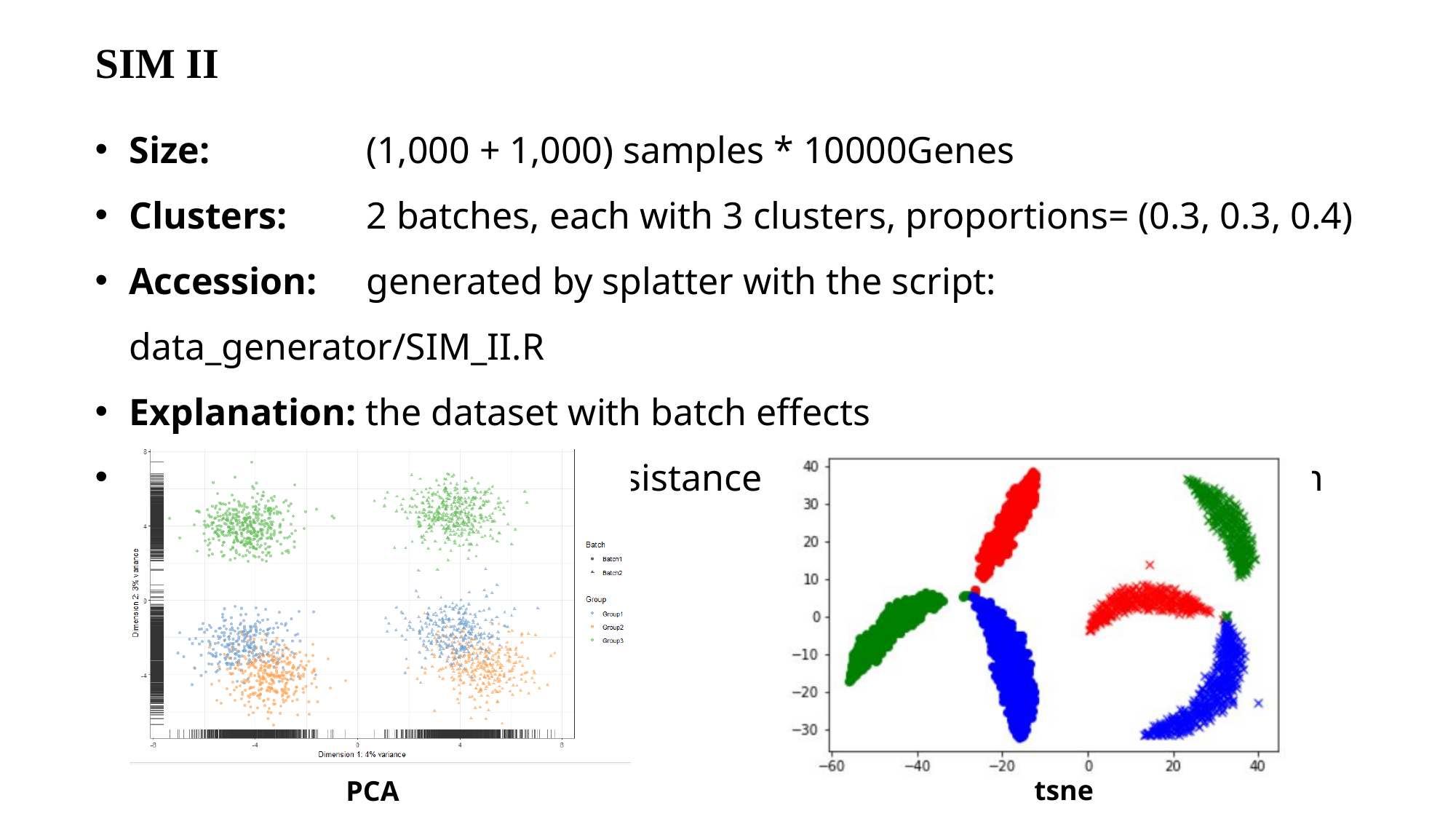

SIM II
Size:		 (1,000 + 1,000) samples * 10000Genes
Clusters:	 2 batches, each with 3 clusters, proportions= (0.3, 0.3, 0.4)
Accession:	 generated by splatter with the script: data_generator/SIM_II.R
Explanation: the dataset with batch effects
Usage:	 to study the resistance to batch effects of the algorithm
tsne
PCA

## Slide 4
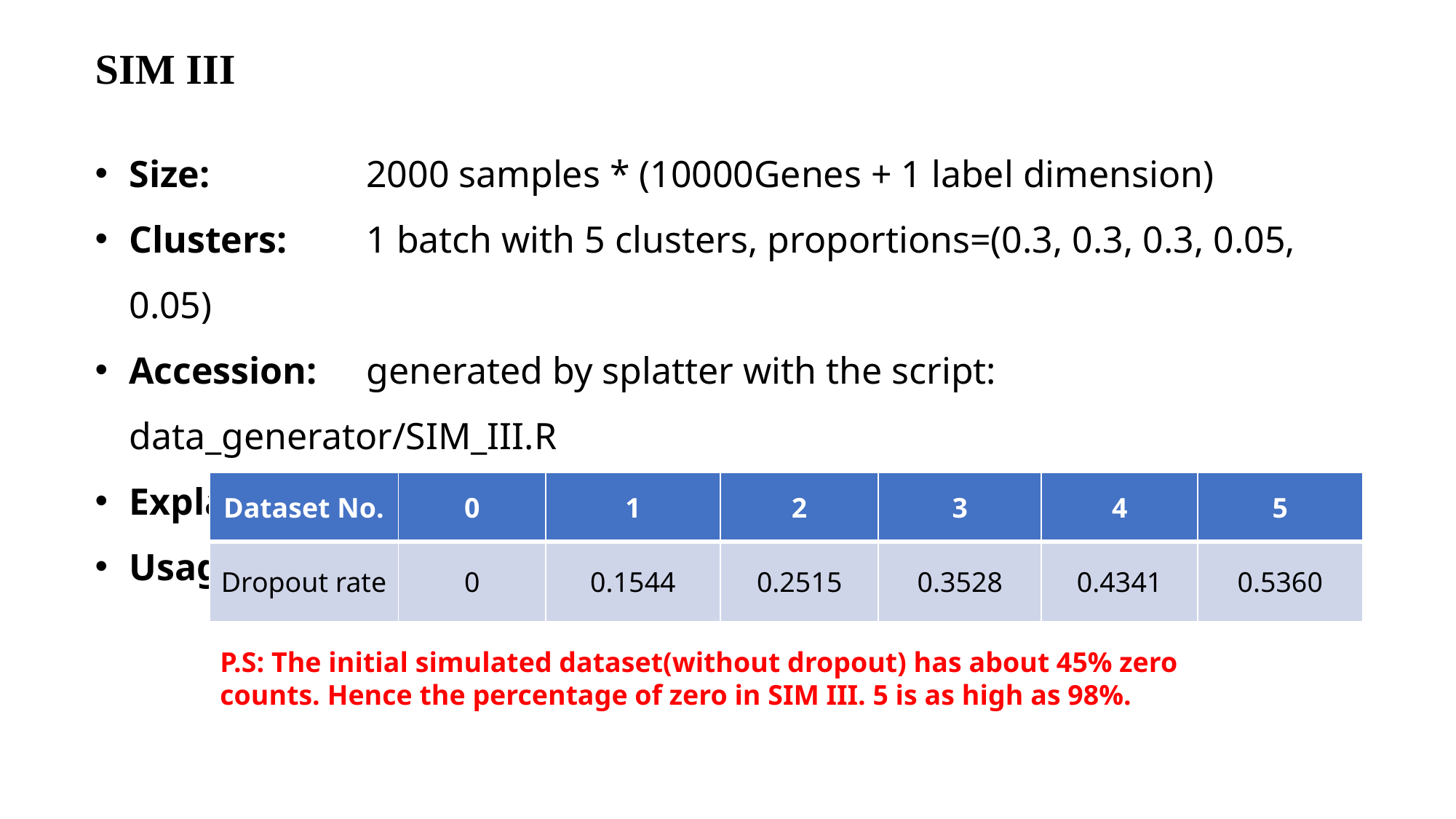

SIM III
Size:		 2000 samples * (10000Genes + 1 label dimension)
Clusters:	 1 batch with 5 clusters, proportions=(0.3, 0.3, 0.3, 0.05, 0.05)
Accession:	 generated by splatter with the script: data_generator/SIM_III.R
Explanation: the datasets with dropout events
Usage:	 to study the resistance to dropout of the algorithm
| Dataset No. | 0 | 1 | 2 | 3 | 4 | 5 |
| --- | --- | --- | --- | --- | --- | --- |
| Dropout rate | 0 | 0.1544 | 0.2515 | 0.3528 | 0.4341 | 0.5360 |
P.S: The initial simulated dataset(without dropout) has about 45% zero counts. Hence the percentage of zero in SIM III. 5 is as high as 98%.

## Slide 5
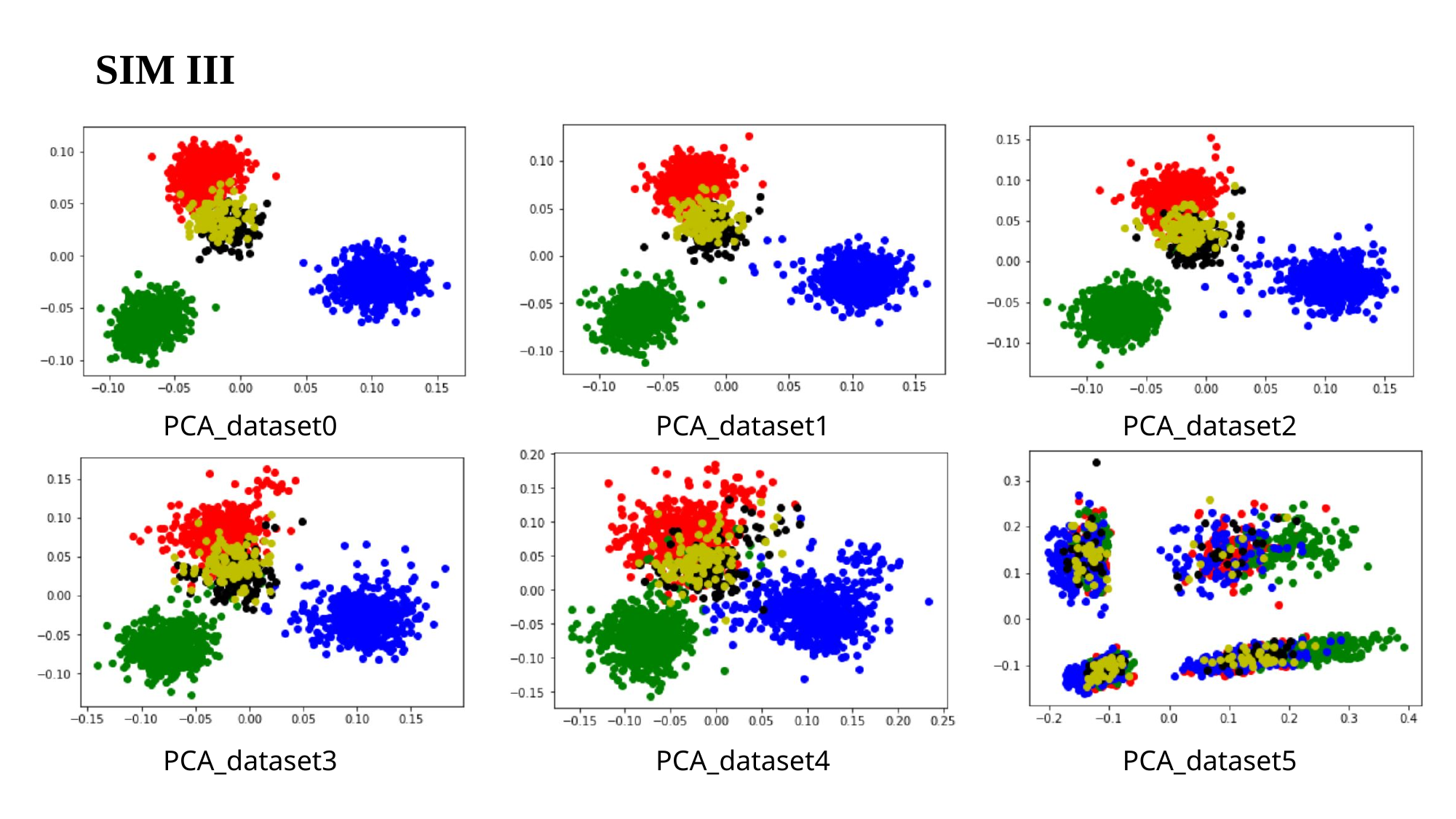

SIM III
PCA_dataset0
PCA_dataset1
PCA_dataset2
PCA_dataset3
PCA_dataset4
PCA_dataset5
